# Genome Assembly of *Shigella flexneri* ATCC 12022, a Quality Control Reference Strain

**DOI:** 10.1128/genomeA.01052-14

**Published:** 2014-10-30

**Authors:** H. E. Daligault, K. W. Davenport, T. D. Minogue, K. A. Bishop-Lilly, S. M. Broomall, D. C. Bruce, P. S. Chain, S. R. Coyne, T. Freitas, K. G. Frey, H. S. Gibbons, J. Jaissle, C.-C. Lo, L. Meincke, A. C. Munk, C. L. Redden, C. N. Rosenzweig, S. L. Johnson

**Affiliations:** a Los Alamos National Laboratory (LANL), Los Alamos, New Mexico, USA; b Diagnostic Systems Division (DSD), United States Army Medical Research Institute of Infectious Diseases (USAMRIID), Fort Detrick, Maryland, USA; c Naval Medical Research Center (NMRC)-Frederick, Fort Detrick, Maryland, USA; d Henry M. Jackson Foundation, Bethesda, Maryland, USA; e United States Army Edgewood Chemical Biological Center (ECBC), Aberdeen Proving Ground, Maryland, USA

## Abstract

*Shigella flexneri* causes shigellosis, severe and potentially life-threatening diarrhea, and accounts for 18% of shigellosis cases in the United States. Here, we present the 4.51-Mbp genome assembly of *S. flexneri* ATCC 12022, a quality control and reference strain, in 10 scaffolds.

## GENOME ANNOUNCEMENT

*Shigella flexneri* is a Gram-negative anaerobic rod and the causative agent of bacterial diarrhea and dysentery in humans. The principal disease, shigellosis, can be caused by as few as 10 to 100 organisms (1). Other clinical presentations of *S. flexneri* infection include hemolytic uremic syndrome (HUS), bacteremia, and toxic megacolon (2). According to the U.S. Centers for Disease Control, *S. flexneri* causes approximately 18% of shigellosis cases in the United States. For this project we sequenced the genome of *S. flexneri* ATCC 12022 (CDC 3591-52), a serovar 2b strain that is used in a variety of quality control tests.

High-quality genomic DNA was extracted from a purified isolate using a QIAgen Genome Tip-500 at the USAMRIID-Diagnostic Systems Division (DSD). Specifically, a 100-mL bacterial culture was grown to stationary phase and nucleic acid extracted per the manufacturer’s recommendations. Sequence data for the draft genome was generated using a combination of Illumina and 454 technologies (3, 4). For the genome, we constructed and sequenced an Illumina library of 100-bp reads at 320-fold genome coverage and a separate long insert paired-end library (6,577 ± 1,644-bp insert, 15-fold genome coverage, Roche 454 Titanium platform). The two libraries were assembled together in Newbler (Roche, version 2.6) and the consensus sequences computationally shredded into 2-kbp overlapping fake reads (shreds). The raw reads were also assembled in Velvet and those consensus sequences computationally shredded into 1.5-kbp overlapping shreds (5). Draft data from all platforms were then assembled together with Allpaths and the consensus sequences computationally shredded into 10-kbp overlapping shreds (6). We then integrated the Newbler consensus shreds, Velvet consensus shreds, Allpaths consensus shreds, and a subset of the long-insert read pairs using parallel Phrap (High Performance Software, LLC, version SPS-4.24). Possible misassemblies were corrected and some gap closure accomplished with manual editing in Consed (7–9).

Automatic annotation of the *S. flexneri* ATCC 12022 genome utilized an Ergatis based workflow at LANL with minor manual curation. The final assembly of 4,513,854-bp (50.7% G+C content) is in 207 contigs placed into 10 scaffolds and includes 4,725 coding, 12 rRNAs, and 95 tRNA sequences.

### Nucleotide sequence accession number.

The annotated genome assembly of *S. flexneri* ATCC 12022 is publicly available in GenBank as JPPN00000000.
